# Space-induced bifurcation in repression-based transcriptional circuits

**DOI:** 10.1186/s12918-014-0125-z

**Published:** 2014-11-08

**Authors:** Amanda Lo Van, Hedi A Soula, Hugues Berry

**Affiliations:** INRIA, 56 Blvd Niels Bohr, Villeurbanne, 69603 France; LIRIS, Université de Lyon, UMR 5205 CNRS-INSA, Villeurbanne, 69621 France; Université de Lyon, Inserm UMR1060, Villeurbanne, 69621 France

**Keywords:** Gene expression, Repressilator, Spatial dynamics, Spatial organization

## Abstract

**Background:**

Albeit the molecular mechanisms of gene expression are well documented, our understanding of their dynamics is much less advanced. Recent experimental evidence has revealed that gene expression might be accurately organized in space, with several molecular actors localized to specific positions in the cell. However, the influence of this spatial localization on the dynamics of gene expression is unclear. This issue is also central in synthetic biology, where one usually considers the spatial localization in the cell of the genes of the inserted synthetic construct as irrelevant for its temporal dynamics.

**Results:**

Here, we assessed the influence of the spatial distribution of the genes on the dynamics of 3-gene transcriptional ring networks regulated by repression, i.e. repressilator circuits, using individual-based modelling to simulate their dynamics in two and three space dimensions. Our simulations suggest that variations of spatial parameters – namely the degree of demixing of the positions of the gene or the spatial range of the mRNA and proteins (i.e. the typical distance they travel before degradation) – have dramatic effects by switching the dynamical regime from spontaneous oscillations to a stationary state where each species fluctuates around a constant value. By analogy with the bifurcations arising from the variation of kinetic parameters, we referred to those transitions as space-induced bifurcations.

**Conclusions:**

Taken together, our results strongly support the idea that the spatial organization of the molecular actors of transcriptional networks is crucial for the dynamics of gene expression and suggest that the spatial localization of the synthetic genes in the cell could be used as an additional toggle to control the dynamics of the inserted construct in synthetic biology experiments.

**Electronic supplementary material:**

The online version of this article (doi:10.1186/s12918-014-0125-z) contains supplementary material, which is available to authorized users.

## Background

In a seminal paper published more than 50 years ago [1], F. Jacob and J. Monod proposed a generic mechanism for protein synthesis in cells, whereby a DNA gene produces a messenger RNA molecule (mRNA) which is then used to produce the corresponding protein. They also described how this gene expression process is controlled by cytosolic macromolecules called repressors, that stop the expression of a given gene by binding to it. Since then, it was discovered that repressors are actually proteins (or sometimes RNAs) produced by other genes and that positive effectors (activators) also exist [2]. Fifty years of molecular studies have unravelled the complexity of the underlying molecular machinery [3,4], but the existence and biological significance of such repression-based transcription systems have been thoroughly confirmed.

The design, insertion in cells and theoretical analysis of small synthetic transcriptional regulation networks have proven to be very useful to our understanding of the temporal dynamics of gene networks. From a purely theoretical standpoint [5-7], repression-based transcriptional regulation loops (i.e. ring networks), are generically expected to exhibit bistability with hysteresis (even numbers of genes) or a regime of spontaneous periodic oscillations (odd numbers of genes). Accordingly, insertion of 2-gene repression-based synthetic loops in bacteria indeed may give rise to bistable dynamics [8], whereas 3-gene repression-based synthetic loops inserted into living bacteria are capable of spontaneous oscillations with a very long period (more than 2.5 hours, i.e. twice the cell doubling time) [9]. In both articles [8,9], the construction of the synthetic networks and their insertion and study in living bacteria were accompanied by a short theoretical study explaining why the observed dynamics were to be expected. In both cases, the complex dynamics of interest arose in the models via a bifurcation from a unique stable steady state: varying one kinetic parameter, the model predicts the occurrence of a saddle-node bifurcation supporting bistability (2 genes) or a Hopf bifurcation explaining the appearance of a limit cycle and its spontaneous oscillations (3 genes). Interestingly, both models consisted of ordinary differential equations (ODE) that assume mass action kinetics [10] for all the reactions. This corresponds to a strong assumption about the internal medium of the cell that is supposed to be dilute, perfectly-stirred and spatially homogeneous. Beyond its importance for switch-like or oscillator circuits, this view in fact has deep impact in the whole field of synthetic biology. When inserting synthetic gene network constructs in chassis cells, synthetic biologists usually do not consider that the spatial localisation of the corresponding plasmids or of the insertion points on the chromosome is an important parameter for the temporal dynamics of the synthetic construct. Whereas this viewpoint seems reasonable if the hypothesis of a perfectly-mixed internal space is valid, it should be questioned if spatial homogeneity is violated.

Actually, the traditional view of the interior of the cell as a perfectly-stirred and spatially homogeneous medium, where the concentration of each reactant would be identical wherever one measures it inside the cell, has increasingly been challenged by recent results, especially in bacterial cells. First, recent advances in the measurement of single particle trajectories inside living cells have unravelled that the bacterial cytoplasm is a complex, extremely crowded and dense medium that strongly affects molecule mobility in a spatially non-homogeneous way [11-15]. Therefore, due to the intrinsic physical nature of the cytoplasm, mobility inside the cytoplasm may not guarantee perfect mixing and homogeneous spatial distribution of its constituents. Recent experimental results have shown that the position of chromosomes in the nucleus of eukaryotic cells or the location of the genes on the bacterial chromosome are not random or perfectly-stirred but self-organized to sit on specific locations forming spatial maps that are stable over long time scales [16,17]. Even in bacteria, transcription is believed to be organized spatially, with the major molecular actors assuming stable and defined intracellular locations [18-20].

Despite this accumulating evidence of the localization of the elements of transcriptional regulation networks inside the cell, the influence of spatial properties on the temporal dynamics of gene expression remains to be fully described. Indeed, in the simplest instance of classical biochemical reactions, the impact of spatial localization has only recently received attention in the case of enzyme complexes [21-23] and in the case of signalling clusters in membrane domains [24-26]. All these results show that spatial correlation strongly modifies the apparent chemical affinity involved in the pathway both for the transient and equilibrium behaviors. Additionally, some evidence suggest that this depends strongly on the actual diffusion values.

In this paper, we use computer simulations to explore if and how the localization of the genes can influence the dynamics of small repression-based transcriptional regulation ring networks. We focus on repression-based transcriptional regulation loops composed of three genes, i.e. repressilators. Using stochastic spatially-explicit individual-based computer simulations, we find that the localization in space of the genes is of crucial importance to the dynamics of the system since it controls even the global dynamics regime, i.e. whether the system fluctuates around stationary values or exhibits spontaneous oscillations. We show that when parameters related to the spatial organization of the genes change, the repressilator undergoes a sharp transition between the oscillatory and stationary regime. Effective control spatial parameters include the degree of demixing of the gene locations or the spatial range (i.e. the typical distance travelled before degradation) of the mRNA and proteins. Since this transition is very similar to the bifurcations along the kinetics parameters that are usually evoked to explain the appearance of the oscillatory regime, we refer to it as a space-induced bifurcation. Our results therefore unravel the importance of spatial properties in the dynamics of transcriptional regulation networks. Moreover, they suggest that the spatial localisation of the synthetic genes in the cell could be used as an additional toggle to control the dynamics of the inserted construct in synthetic biology experiments.

## Methods

Our objective is to study the dynamics in time and intracellular space of a generic transcriptional circuit in a bacterial cell like *E. coli*. Extensively detailed realistic modelling of bacteria (whole-cell models), with experimentally-derived values for most rate constants, realistic cell space volume, protein size and diffusion coefficients, and interaction with the metabolism, starts to be accessible to today computer power [27-30]. However, those models bear the limitation that many of the parameters still lack experimentally-derived values. Moreover, their computational cost still forbids to use them for thorough exploration of the parameter space with reasonable statistical significance, which is precisely the objective of the present work. We therefore opted for simpler models and restricted our focus to the study of the major processes implicated in transcriptional regulation (and described in the seminal Jacob and Monod paper [1]) rather than taking into account extensive details of the cell intracellular machinery.

### Gene expression model

**Figure 1:**
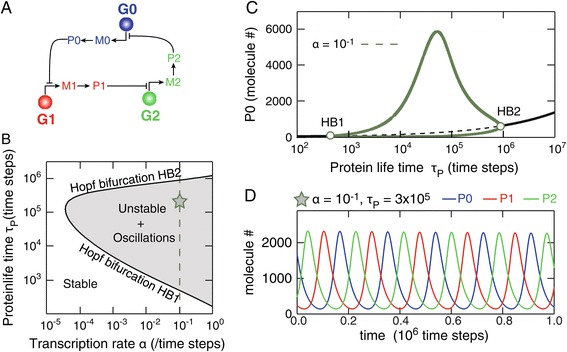
**Mass action kinetics predict that 3-gene repression-based transcriptional circuits are generic oscillators.** **(A)** A generic transcriptional circuit with three components and circular repression. *G*_*i*_, *M*_*i*_ and *P*_*i*_ refer to the gene, mRNA and protein, respectively, of components *i*∈{0,1,2}. **(B)** Bifurcation diagram of the mass action kinetics approximation of the model of **(A)** ((2)) in the (*τ*_*P*_−*α*) plan. Two Hopf-Bifurcation branches (full lines) separate a region of spontaneous oscillatory behavior (grey area) from stable steady state behavior. **(C)** Bifurcation diagram along *τ*_*P*_ for *α*=0.1 (green dashed line in **B**). Full thick black lines locate stable steady-states, the dashed black line locates unstable steady-states, and thick green lines show minimal and maximal values (envelope) of limit cycle oscillations. **(D)** Spontaneous oscillations of the three proteins for *α*=0.1 and *τ*_*P*_=3×10^5^ (green star in **B**). All other parameters were according to the standard set defined in the Methods section.

We considered a generic transcriptional circuit consisting of three genes *G*_*i*_, *i*∈{0,1,2} assembled in a circular circuit, or ring network, so that gene *i* represses the expression of gene *i*+1 (Figure 1A). Each gene *G*_*i*_ is spontaneously (constitutively) transcribed into mRNA *M*_*i*_, that is in turn spontaneously translated to protein *P*_*i*_. Each gene features two binding sites for its repressor protein set so as to achieve cooperative binding, i.e. the binding affinity is larger when the gene is already bound to a single protein. According to the circular circuit of Figure 1A, each gene *G*_*i*_ can bind the protein product of gene $\bar {i}=(i+2)\text {mod}(3)$ and single- or double-binding of a gene shuts-off transcription (repression). These processes can be described according to the following reaction schemes:

(1) $$ \forall i\in\{0,1,2\}, \left\{ \begin{array}{r@{}cl} G_{i}& {\overset{\alpha}{\longrightarrow}}&G_{i}+M_{i}\\ M_{i}& {\overset{\beta}{\longrightarrow}}&M_{i}+P_{i}\\ M_{i}& {\overset{1/\tau_{M}}{\longrightarrow}}&\emptyset\\ P_{i}& {\overset{1/\tau_{P}}{\longrightarrow}}&\emptyset\\ G_{i}+P_{\overline{i}}& {\overset{{k}_{\text{on}}}{{\underset{{k}_{\text{off}}}{\rightleftharpoons}}}} &G_{i}^{\star}\\ G_{i}^{\star}+P_{\overline{i}}& {\overset{{k}_{\text{on}}}{{\underset{{k}_{\text{off2}}}{\rightleftharpoons}}}} &G_{i}^{\star\star} \end{array} \right.  $$

where $\overline {i}=(i+2)\text {mod}(3)$; *α* and *β* are the transcription and translation rates, respectively; *τ*_*M*_ and *τ*_*P*_ are the lifetimes of mRNA and proteins; $G_{i}^{\star }$ and $G_{i}^{\star \star }$ denote the singly- and doubly-bound genes, respectively and *k*_on_, *k*_off_, *k*_off2_ the corresponding reaction rate constants. Note that to keep the model as simple as possible, the values of those constants were chosen identical for all genes *i*.

### Mass action kinetics approximation

According to classical mass action kinetics, the reactions of (1) are transcribed in the following system of ordinary differential equations (ODEs):

(2) $$ \forall i\in\{0,1,2\}, \left\{ \begin{array}{r@{}l} \frac{\mathrm{d}G_{i}}{\mathrm{d}t}&=-k_{\text{on}}G_{i}P_{\overline{i}}+k_{\text{off}}G_{i}^{\star}\\ \frac{\mathrm{d}M_{i}}{\mathrm{d}t}&=\alpha G_{i}-M_{i}/\tau_{M}\\ \frac{\mathrm{d}P_{i}}{\mathrm{d}t}&=\beta M_{i}-P_{i}/\tau_{P}-k_{\text{on}}P_{i}\left(G_{\overline{i-1}}+G^{\star}_{\overline{i-1}}\right)+k_{\text{off}}G_{\overline{i-1}}^{\star}+k_{\text{off2}}\left(G_{\overline{i-1}}^{T}-G_{\overline{i-1}}-G_{\overline{i-1}}^{\star}\right)\\ \frac{\mathrm{d}G_{i}^{\star}}{\mathrm{d}t}&=k_{\text{on}}P_{\overline{i}}\left(G_{i}-G_{i}^{\star}\right)-k_{\text{off}}G_{i}^{\star}+k_{\text{off2}}\ensuremath{\left({G_{i}^{T}}-G_{i}-G_{i}^{\star}\right)}\\ \end{array} \right.  $$

where we used mass conservation for the different bound fractions of the genes, i.e. $G_{i}+G_{i}^{\star }+G_{i}^{\star \star }={G_{i}^{T}}=\text {constant}$.

#### Parameter values and numerics

Numerical integration (4th order Runge-Kutta method) and bifurcation analysis were performed with Xppaut (available at www.math.pitt.edu/~bard/xpp/xpp.html). To keep the model as generic as possible, we expressed time in multiples of the integration time step (*Δt*=1), thus yielding reaction rate constants expressed as inverse of time steps. The bifurcation diagram was explored along the protein lifetime *τ*_*P*_ and the transcription rate *α* (Figure 1B-C). Those parameters were thus varied over several orders of magnitude. The values of the translation rate *β* and the mRNA lifetime *τ*_*M*_ were set so as to ensure realistic copy numbers of proteins and mRNAs. mRNA copy number in single *E. coli* cells was quantified between <0.1 and 5 [31] or even up to 50 for strongly expressed promoters like the P_*lac*_ promoter under strong induction [32]. Protein copy numbers vary over a wide range, from 10 to 10^5^ [31,33], resulting in a protein/mRNA copy number ratio that is roughly between 10^2^ to 10^4^ [31]. In our model, the range of mRNA/Protein copy number *M*_*i*_/*P*_*i*_ is given by the product of parameters *τ*_*P*_*β*. Since *τ*_*P*_ was varied between 10^3^ and 10^6^ in the bifurcation diagram, we set *β*=0.1. In *E. coli*, the typical lifetime of mRNAs (minutes or less) is much smaller than that of proteins (larger than one cell cycle), especially fluorescent reporter proteins (like GFP) [31,34]. Considering the range of values over which the protein lifetime *τ*_*P*_ was varied, we fixed *τ*_*M*_=50 time steps.

The affinity of DNA regulation proteins (transcription factors) for their specific DNA site is also variable, but typically reported values are between 0.5–1.0 nM [35] and several tens (to few hundreds) of nM [36,37]. Given a total *E. coli* cell volume of 3 fl [38], we set *k*_on_=10^−5^ per molecule per time step and *k*_off_=10^−3^ and *k*_off2_=10^−5^ per time step. This corresponds to affinities of 55 and 0.55 nM for the free (unbound) and singly-bound genes, respectively. Finally, we considered that each of the three genes can be present in the bacterial cell in multiple copies (i.e. ${G_{i}^{T}}>1$). Indeed, even with low copy number plasmids (as used in [9]), each gene is introduced in 3−4 to 10 copies [39]. Therefore, unless otherwise specified the number of copies per gene type was set to ${G_{i}^{T}}=5$ (∀*i*∈{0,1,2}).

### Individual-based simulation of the spatial dynamics

A spatially-explicit stochastic individual-based (Monte-Carlo) simulator of the set of biochemical reactions given by (1) was implemented as a lattice-based algorithm programmed in C. Boundary conditions were periodic. Each of the 3 gene types were present in *G*_*T*_ copies.

#### Spatial configuration of the genes

**Figure 2:**
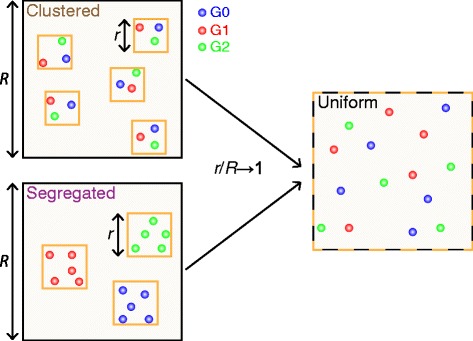
**Spatial configurations of the genes in the individual-based model.** This scheme illustrates the case *G*_*T*_=5 in 2D. In the *uniform* configuration, the 15 gene copies are positioned at independent randomly-chosen lattice sites, whereas in the *clustered* and *segregated* configurations, we start by positioning internal boxes of linear size *r* at random positions in the lattice, and place the gene copies inside the boxes. In the *clustered* configuration, each box contains one copy of each gene type *i*∈{0,1,2}, randomly positioned, while in the *segregated* configuration, each box receives all the *G*_*T*_ copies of gene 0,1 or 2, positioned at randomly chosen locations. When the internal box size *r* tends to the lattice size *R*, both clustered and segregated configurations converge to the uniform configuration.
- In the *clustered* configuration, we first positioned *G*_*T*_ internal boxes of linear size *r* on the lattice. One copy of each gene *i*∈{0,1,2} per box was then positioned at a randomly chosen location inside the box.
- In the *segregated* configuration, we first positioned 3 internal boxes of linear size *r* on the lattice. Each of the 3 boxes received all of the *G*_*T*_ copies of gene 0,1 or 2, positioned at randomly chosen locations inside the box.

The initial locations of the 3*G*_*T*_ genes were set according to one of the following three scenarios (Figure 2):

- In the *uniform* configuration, the 3*G*_*T*_ gene copies were positioned at independent randomly-chosen lattice sites (with uniform probability).

When the box size *r* converges toward the lattice size *R*, the clustered and segregated configurations are closing to the uniform one. The ratio *r*/*R* can thus be used to quantify the amount of demixing (segregation or clustering): vanishing demixing for *r*/*R*→1 and strong demixing for *r*/*R*→0. The thus defined gene locations were kept constant during the simulation, i.e. the genes were immobile.

#### Simulation algorithm

At the beginning of each simulation, we place 10 mRNA and 10^4^ proteins of each type *i* at random positions (with uniform distribution) on the lattice. At each Monte-Carlo time step (*Δt*=1), the reaction events (transcription, translation, binding and unbinding) are simulated according to the following schedule:

1. Each free (unbound) gene copy transcribes a new mRNA molecule *M*_*i*_ with probability *α*.
2. Every mRNA molecule *M*_*i*_ can be degraded (with probability 1/*τ*_*M*_) or can translate a new protein *P*_*i*_ (with probability *β*≤1−1/*τ*_*M*_). If not degraded, each mRNA then undergoes a random walk step (see below).
3. Each free (unbound) protein is degraded with probability 1/*τ*_*P*_ or undergoes a random walk step (with probability 1−1/*τ*_*P*_).
4. Whenever a free protein *P*_*i*_ shares its lattice site with a free target gene $G_{\overline {i-1}}$ or singly-bound target gene $G^{\star }_{\overline {i-1}}$, binding occurs with probability *k*_on_.
5. Bound gene-protein complexes that were formed before the current time step can unbind depending on their occupancy status, i.e. with probability *k*_off_ or *k*_off2_, if singly- or doubly-bound, respectively.

At each random walk step, the walker changes its current location with probability *p*_move_, moving to one of its 4 (2D) or 6 (3D) randomly-chosen nearest neighbors (with uniform probability). This corresponds to diffusion coefficient *D*=*p*_move_/4 (2D) or *D*=*p*_move_/6 (*latticespacing*)^2^/time step (3D). Note that all diffusive molecules (mRNA and proteins) have identical diffusion coefficient.

#### Parameter values and numerics

The internode distance of the space lattice was set to *Δx*=1 (arbitrary units, a.u.) and the lattice size was set to *R*×*R*=400×400 a.u.^2^ (2D) or *R*×*R*×*R*=60×60×60 a.u.^3^ (3D). Time was expressed in numbers of Monte-Carlo (MC) time steps. Regarding the values of the other parameters, our goal here was to compare with the dynamics predicted by mass action kinetics. Therefore, unless otherwise specified, the standard set of parameters in the individual-based simulations was taken identical to the mass action kinetics model above, i.e. *G*_*T*_=5 copies, *τ*_*M*_=50 MC time steps, *τ*_*P*_=10^3^ MC time steps; probability rates in (MC time steps)^−1^: *α*=*β*=0.10, *k*_off_=10^−3^ and *k*_off2_=10^−5^. Note that the value of *k*_on_ for individual-based simulations is expected to differ from the value used in mass action law kinetics. Indeed in the former, *k*_on_ is a reaction probability rate upon reactant encounter in space, while in the latter *k*_on_ is a classical reaction rate constant that, in addition to the reaction probability upon reactant encounter also accounts for reactant encounter probability. Taking the size of the reaction volume into account (400×400 or 60×60×60) we estimated that *k*_on_=1 per MC time step per encounter in individual-based simulations was comparable with the value used in the mass action kinetics model. Finally, the standard value of the movement probability was set to *p*_move_=1.0, yielding diffusion coefficients *D*=0.250 or 0.167 a.u.^2^/MC time step in 2D and 3D, respectively.

### Quantifying gene expression dynamics

To analyze the resulting simulation dynamics, we used the first zero crossing of the autocorrelation function. For each protein time series *P*_*i*_(*jΔt*), *j*∈{0,1,…,10^7^} resulting from the individual-based simulations, we first sliced each protein time series into 100 segments of identical length *l*=10^5^ time steps. On each window, the autocorrelation function was computed as

$$ACF(\tau)=\frac{\mathbb{E}\left[\left(P_{i}(j\Delta t+\tau)-\overline{P_{i}}\right)\left(P_{i}(j\Delta t)-\overline{P_{i}}\right)\right]}{\sigma^{2}_{P_{i}}} $$

where $\mathbb {E}[]$ denotes expectation over the time points, $\overline {P_{i}}$ is the mean of the time series over the window, $\sigma ^{2}_{P_{i}}$ its variance, *j*∈ [ 0,*l*−*τ*] and the lag time *τ*∈ [ 0,*l*−1]. We then averaged the *ACF*(*τ*) values across each segment of the time series. The first zero crossing of the thus averaged *ACF* (FZCA) is the smallest value *τ*_0_ such that *ACF*(*τ*_0_)=0. One FZCA was computed for each protein time series resulting from the individual-based simulation. For each simulation run, we retained the minimal value among the three estimated values (one per protein type). Note that the first 2×10^6^ MC time steps of each time series were rejected to discard transient behaviors.

## Results

Here, we study a generic transcriptional circuit consisting of three genes *G*_*i*_, *i*∈{0,1,2} forming a transcriptional ring network of repression: *G*_0_ represses the expression of *G*_1_, *G*_1_ represses *G*_2_ and *G*_2_ closes the loop by repressing *G*_0_ (Figure 1A). Such repression-based transcriptional circuits are sometimes referred to as “repressilator” circuits in the literature [9], because circuits made of odd numbers of negative interactions are, generically, potential spontaneous oscillators [6,7]. In agreement with the model proposed by Jacob and Monod [1], we assume that each gene *G*_*i*_ is active in the absence of bound protein, thus producing mRNA transcripts *M*_*i*_ (at rate *α*) then the corresponding protein *P*_*i*_ (rate *β*). Each gene *G*_*i*_ can bind up to two copies of its repressing protein, with cooperative binding. Once bound, genes stop transcribing mRNAs. mRNAs and proteins have finite life-times *τ*_*M*_ and *τ*_*P*_ respectively. See Methods for further details, in particular the set of elementary reaction schemes (1) that describes the system.

In the following, we study this generic repression-based transcriptional circuit with two modelling approaches: the traditional mass action kinetics, that assume perfectly-stirred conditions (infinitely fast mixing) thus neglecting the effects of spatial fluctuations and stochastic individual-based (Monte-Carlo) simulations that explicitly take into account spatial fluctuations.

### Mass action kinetics predict spontaneous oscillations

The traditional approach in biochemistry to model the kinetics of such generic repression-based transcriptional circuit is based on the theory of elementary chemical reaction kinetics, usually referred to as “Mass action kinetics” [10]. Mass action kinetics are mean-field approximations assuming that the reaction medium is dilute, perfectly-stirred and spatially homogeneous. Under those assumptions, one considers that the local fluctuations of reactant concentration (induced by e.g. the reaction itself) can be neglected and replaces local reactant concentrations by their average values over a large spatial domain (usually the whole reaction volume). For the generic repression-based transcriptional circuit studied here, mass action kinetics yield the system of 3×4 coupled Ordinary Differential Equations (ODEs) shown as (2).

Figure 1B-C shows a bifurcation analysis of these equations. In the two-dimensional parameter space defined by the transcription rate *α* and the protein lifetime *τ*_*P*_, the system presents two regions delineated by two Hopf bifurcation branches. Outside the region enclosed by the Hopf bifurcation branches, (2) has a single, stable steady-state (the white region in Figure 1B). All the reactants are therefore predicted to converge at long times to stationary values. Inside the grey region of Figure 1B, this steady-state loses its stability and this stability loss is accompanied by the birth of a stable limit cycle (Figure 1C). The points in the parameter space where the steady-state changes stability and the limit cycle appears constitute the Hopf Bifurcation branches. The dynamics in the region delimited by the Hopf bifurcation branches thus consists in spontaneous oscillations where all the mRNA and protein species oscillate in time (Figure 1D). Because of the cyclic cooperative repression, protein oscillations are two-by-two anti-synchronized: protein *P*_*i*_ reaches its peak values when *P*_*i*−1_ is minimal.

### Individual-based simulations predict strong dependence on the spatial locations of the genes

Mass-action kinetics, albeit widely used, is actually just one methodology among several others to model the dynamics of the reaction set (1). To evaluate the possible effects of the spatial extension of these reactions, one has to employ alternative modelling methodology. Spatially explicit individual-based simulations (Monte-Carlo simulations, see Methods) explicitly describe the diffusive motion of each individual mRNA and protein molecules and emulate the reaction steps of (1) as stochastic processes whenever the concerned reactants encounter in space along their respective random walk (diffusion). Just like for the mass action kinetics, each gene type *i*∈{0,1,2} is present in *G*_*T*_ copies. In agreement with previous models of the influence of diffusion on gene expression [40,41], we assumed that the amplitude and speed of the gene displacements in space can be neglected compared to the mRNA and protein, so in our simulations, the genes are immobile and only the mRNAs and proteins move via Brownian motion (with identical diffusion coefficients).

To set the positions of the 3*G*_*T*_ genes in space, we compared three scenarios that correspond to various degrees of mixing (Figure 2):

- The *uniform* configuration corresponds to a perfect mixing of the genes: all 3*G*_*T*_ gene copies are positioned at independent randomly-chosen locations.
- The *clustered* configuration corresponds to a first case of demixing: *G*_*T*_ gene triplets composed of one *G*_0_, one *G*_1_ and one *G*_2_ gene are restricted inside non-overlapping subregions of space
- The *segregated* configuration illustrates a different case of demixing: the 3 gene types *i*∈{0,1,2} are segregated into 3 subregions of space, each subregion containing all the *G*_*T*_ copies of a given type.

The degree of demixing of the genes can be continuously adjusted by setting the ratio *r*/*R* between the size of the local segregation subregions *r* and that of the total reaction space *R*. Demixing is strong for *r*/*R*→0 (either for the clustered or segregated scenario) but disappears when *r*/*R*→1.

**Figure 3:**
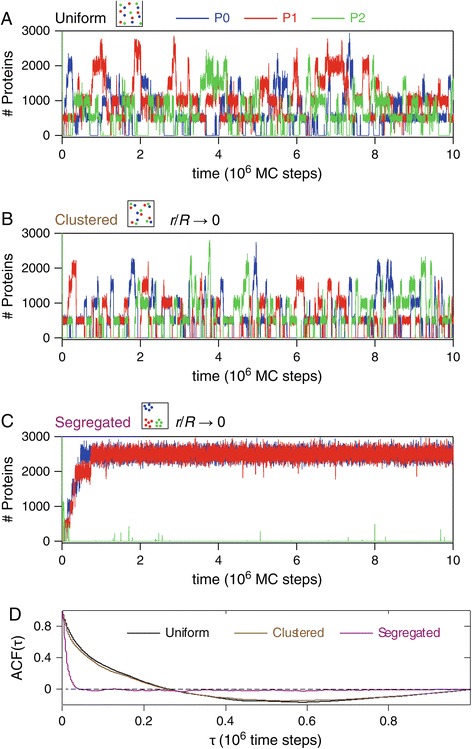
**The spatial configuration of the genes may alter the dynamical regime (2D simulations).** These time courses of the total number of proteins (*P*_0_ in blue, *P*_1_ red and *P*_2_ green) in the reaction space were obtained using spatially-explicit stochastic individual-based simulations in 2D. The spatial configuration of the genes corresponded to the **(A)** uniform, **(B)** clustered (*r*=3) or **(C)** segregated (*r*=3) scenarios of Figure 2. The autocorrelation function for each of these three spatial configurations is shown in **(D)** (see Methods for calculation of the autocorrelation function). Size of the spatial domain *R*=400, *G*_*T*_=5 copies. All other parameters were according to the standard set defined in the Methods section.

Figure 3A shows the time courses of the three protein types during a typical simulation of the individual-based stochastic model with uniform gene configuration in 2D. Obviously, the time series have highly noisy aspects since in these simulations, both the occurrence of reactant encounter and the reaction realization upon encounter of the reactants are random processes. The oscillatory nature of the time series is however apparent beyond the noise. The peak protein number roughly agrees wight the predictions of the mass action kinetics with identical parameters (i.e. between 1,000 and 3,000 copy numbers; compare with Figure 1D). The protein peaks still show a certain level of two-by-two anti-synchronization, although it is much less strict than in the deterministic version (i.e. *P*_2_ peaks most often coincide with low levels of *P*_0_ but this is not systematic anymore). To quantify the oscillatory trend, we computed the autocorrelation (*ACF*) function of the time series (Figure 3D). In the uniform configuration, the autocorrelation function has a shape typical of oscillatory time series. It slowly decreases to its first zero crossing, after which it becomes negative and proceeds to oscillate. The first zero crossing of the *ACF* (FZCA) occurs at a delay of 0.264×10^6^ MC times steps. This large value actually defines the period of the oscillations in Figure 3A. Again this value is similar to the period predicted by mass action kinetics for the same parameter values (Figure 1D). Moreover, visual inspection of the time series for the clustered configuration with strong demixing (small *r*/*R*, Figure 3B) indicates that the dynamics are very similar to the uniform case. Accordingly, the *ACF* in the clustered case hardly departs from the uniform one (Figure 3D) and yields almost identical FZCA (0.260×10^6^ MC time steps). We conclude that for the uniform and clustered gene configurations (even with strong demixing), the individual-based stochastic simulations in 2D show temporal dynamics that are very similar to those predicted by mass action kinetics, even though their salient features are blurred by a high degree of stochasticity.

The results of the 2D individual-based stochastic simulations for the segregated gene configuration with large demixing however show a strikingly different behavior (Figure 3C). After a transient period, protein levels do not show evidence of oscillations any longer but reach a stationary level around which they fluctuate. In some cases, the stationary regime for one of the proteins can even correspond to a stably and completely repressed state (*G*_2_ in the example shown in Figure 3C). As a result, the dynamics consists of two proteins (*P*_0_ and *P*_1_ in the case shown in Figure 3C) fluctuating around a stationary level, while a third one (*P*_2_ in Figure 3C) has vanished. We insist here on the fact that the only difference between the oscillatory regime in Figure 3A-B and the stationary one in Figure 3C are the spatial locations of the 5×3 immobile genes. All the other parameters (rate constants, species densities/concentrations, lifetimes …) are identical. This is a major result of our paper: changing a purely spatial characteristics (here the positions of the genes) is enough to change the system dynamics in a *qualitative* manner (from oscillatory to stationary). Since they do not include spatial characteristics, mass action kinetics cannot account for this kind of global change of dynamics. Even worse, the dynamics illustrated in Figure 3C cannot be predicted at all by mass action kinetics. Indeed, according to mass action kinetics ((2)), the only reachable regime consists in a stationary state in which the stationary quantities of protein *P*_*i*_ are identical ∀*i*∈{0,1,2} (since the kinetic parameters are identical for the 3 genes/mRNA/protein types). In other words, the only accessible stationary state according to mass action kinetics should have *P*_0_(*∞*)=*P*_1_(*∞*)=*P*_2_(*∞*), whereas Figure 3C evidences the existence of a stationary regime where *P*_0_(*∞*)=*P*_1_(*∞*)≫0 and *P*_2_(*∞*)≈0.

To quantify the difference between those two regimes, we found that the first zero crossing of the *ACF* is a very good quantifier. Figure 3D shows the *ACF* for the segregated case of Figure 3C. This *ACF* is typical of stochastic time series fluctuating around a stationary mean: it decreases very rapidly with the autocorrelation delay and is roughly devoid of subsequent oscillations. It is very easy to distinguish it from the *ACF* of the uniform and clustered configuration that are typical of oscillating regimes, with large period. In particular the first zero crossing (*FZCA*) is found much smaller in the stationary segregated configuration (0.041×10^6^ MC time steps) than in the oscillatory uniform or clustered ones (>0.2×10^6^ MC time steps). In the following, the *FZCA* will thus be used as a quantifier to distinguish between stationary regimes (*FZCA* on the order of 10 thousands MC time steps) and oscillating ones (*FZCA* of several hundred thousands MC time steps).

### A bifurcation based on the spatial localization of the genes

The segregated and clustered configurations illustrated in Figure 3 above correspond to high demixing (i.e. *r*/*R*<0.010). We then investigated how the observed effects depend on the degree of demixing.

**Figure 4:**
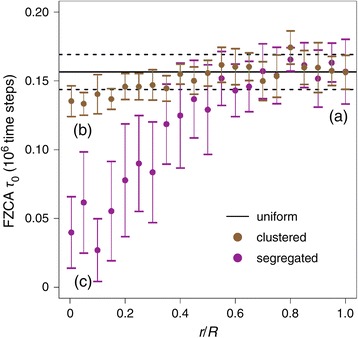
**The switch from oscillations to stationarity as a demixing-induced bifurcation of the segregated configuration (2D simulations).** The average value of the first zero crossing of the *ACF* (*FZCA*) is reported as a function of the degree of demixing *r*/*R*, for uniform (black line), clustered (brown circles) or segregated (magenta circles) gene configurations. For each value of *r*/*R* and each gene configuration, 20 simulations were run (with different realizations of the random choices). Circles (or full horizontal lines) are the average of the 20 resulting *FZCA* while bars (or dashed horizontal lines) show ± 1 s.d. Labels (a), (b) and (c) locate the parameters used to generate the corresponding subpanels of Figure 3. Size of the spatial domain *R*=400, *G*_*T*_=5 copies. All other parameters were according to the standard set defined in the Methods section.

Figure 4 shows the evolution of the *FZCA* averaged over several simulation runs, as the degree of mixing (*r*/*R*) changes. As expected, the three spatial configurations converge to the same regime when the location of the genes is well-mixed (*r*/*R*→1). This corresponds to the oscillatory regime with long period (average *FZCA*≈0.16×10^6^ MC time steps) illustrated in Figure 3A. Therefore, when the positions of the genes are well-mixed, the effect of the gene position vanishes, and all tested spatial configurations tend to the uniform one, corresponding roughly to the prediction of the mass action laws. In the clustered configuration, the average *FZCA* keeps large values whatever the degree of mixing (at least within the range of tested parameters). Therefore the dynamics of the clustered configuration is expected to mostly agree with the prediction from mass action kinetics, yielding slow oscillations for all mixing degrees (period = several hundred thousands of MC time steps).

Here again the segregated scenario yields a very different picture (Figure 4). For large enough mixing (i.e. $r/R\gtrsim 0.5$), the dynamics remain oscillatory, with *FZCA* values that are indistinguishable from the uniform case. For *r*/*R*<0.5, though, the average *FZCA* progressively decreases with increasing demixing, switching from oscillatory values (>0.1 million MC time steps) to values typical of stochastic fluctuations around a stationary state (<0.05 million MC time steps). The corresponding curve actually describes a bifurcation: as demixing crossovers the critical value (*r*/*R*)_*c*_≈0.5, the dynamics undergoes a global (qualitative) change from oscillatory to a stable stationary state. However, the bifurcation parameter here is not a kinetic parameter or density parameter, as usually the case, but a parameter related to the spatial locations of the genes. We refer to this behavior as a space-induced bifurcation.

**Figure 5:**
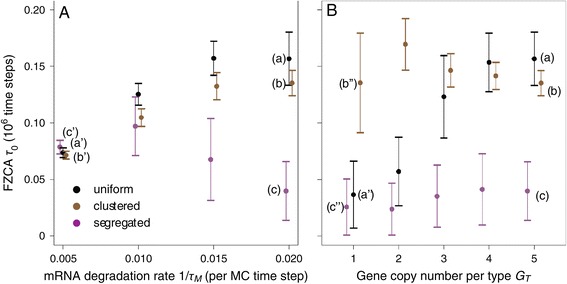
**The space-induced bifurcation depends on kinetics parameters (2D simulations).** The average value of the first zero crossing of the *ACF* (*FZCA*) is reported as a function of the mRNA degradation rate 1/*τ*_*M*_ **(A)** or the number of gene copy *G*_*T*_ **(B)** for uniform (black circles), clustered (brown circles) or segregated (magenta circles) gene configurations. For each value of parameters and each gene configuration, 20 simulations were run. Circles are the average of the 20 resulting *FZCA* and bars ± 1 s.d. In both panels, the horizontal coordinates are shifted for readability. Labels (a), (b) and (c) locate the parameters used to generate the corresponding subpanels of Figure 3, while labels (a’), (b’), (c’) and (a”), (b”), (c”) refer to the examples shown in Additional file 1: Figure S1 (a”), (b’) and (c’) and Additional file 2: Figure S2 (a”’), (b”) and (c”), respectively, in the Supporting Material. Wherever applicable (i.e. except for the uniform configuration), the size of the internal boxes was *r*=3. All other parameters were according to the standard set defined in the Methods section.

In the results of Figures 3 and 4, the mRNA have a finite lifetime of *τ*_*M*_=50 MC steps. With infinite mRNA and protein lifetimes, each protein molecule would be able to reach any gene position given enough time, yielding perfect mixing (albeit possibly slow). One therefore does not expect to observe the spatial effects reported in Figures 3 and 4 when the protein or mRNA lifetimes diverge. Figure 5A shows the *FZCA* values obtained for different values of the mRNA degradation rate (i.e. the inverse of the mRNA lifetime 1/*τ*_*M*_). When the degradation rate vanishes (i.e. the lifetime diverges), the *FZCA* converges to a unique value (around 80 thousands time steps, labels *a*^′^−*c*^′^), independently of the gene spatial configuration. This common regime (see Figure S1 in the Additional file 1) corresponds to an oscillatory regime for all the spatial configurations, even the segregated configuration with high demixing *r*/*R*→0. Therefore, in the limit of very large mRNA lifetimes, the observed regime is in agreement with the predictions of the mass action kinetics: an oscillatory regime that does not depend on the positions of the genes. The space-induced effects unveiled in Figures 3 and 4 start to be significant when the spatial range of the mRNAs *τ*_*M*_*D* or proteins *τ*_*P*_*D* (i.e. the typical average distance travelled before degradation, also referred to as the Kuramoto length [41]) decreases. In other words, the oscillatory regime disappears when the distance between a given segregated gene cluster and the cluster of its repressive genes is too large compared to the mRNA or protein spatial range, thus failing to yield efficient repression.

Finally, Figure 5B shows the *FZCA* values obtained when the copy number of each gene (*G*_*T*_) is varied. In the spatial configurations with large demixing (clustered or segregated configurations), decreasing the number of genes does not qualitatively change the dynamics (see Additional file 1: Figure S1 *b*^″^ and *c*^″^ in the Supporting Material): even with a single copy of each gene type, the segregated configuration keeps stationary dynamics whereas the clustered one maintains its oscillatory regime (albeit with modified waveform). The behavior with the uniform configuration is more complex. By definition, with a unique copy of each gene (*G*_*T*_=1), the uniform and segregated spatial configurations are identical. One the other hand, with standard parameters (i.e. *G*_*T*_=5 copies of each gene), the above results show that the dynamics of the uniform configuration is essentially identical to the clustered configuration. Therefore, in the uniform configuration, one expects a transition from the oscillatory to the stationary regimes when the number of gene copy decreases from 5 to 1. Figure 5B shows that this transition occurs between *G*_*T*_=2 and *G*_*T*_=3. For *G*_*T*_≤2 copies the uniform spatial configuration display the stationary dynamics typical of the segregated configuration (Additional file 2: Figure S2) whereas for *G*_*T*_≥3, the dynamics is oscillatory, similar to the clustered configuration.

Taken together, those results show that with spatially explicit dynamics, the system still displays bifurcations when the kinetic parameters are varied. Spatial parameters thus bring an additional dimension to the bifurcation diagrams, in addition to the kinetic ones.

### Space-induced bifurcation in three dimensions

In the above results, the movement of the reactants in the stochastic individual-based simulations occurred along a two-dimensional spatial domain. Lattice-based simulations of Brownian diffusion in two dimensions are less demanding in terms of computation cost than in three dimensions, thus permitting exploration of the parameter space with reasonable accuracy and sampling. However, compared to two dimensions, Brownian diffusion in three dimensions has fundamentally different properties regarding how space is explored (compactness of the random walk) [42,43]. In this section, we show that the occurrence of space-induced bifurcation is robust to those changes in compacity and is preserved in three dimensional systems.

**Figure 6:**
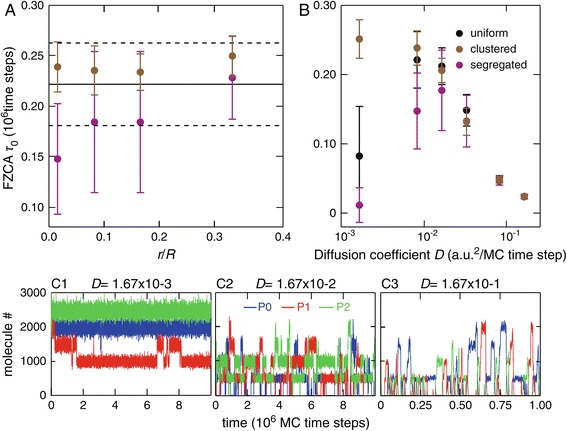
**Space-induced bifurcations are also observed in three dimensions.** The average value of the first zero crossing of the *ACF* (*FZCA*) is reported as a function of the degree of demixing *r*/*R* **(A)** or the diffusion coefficient *D* **(B)** for uniform (black full line), clustered (brown circles) or segregated (magenta circles) gene configurations in 3D. For each value of parameters and each gene configuration, 20 simulations were run. Circles and horizontal full black line are the average of the 20 resulting *FZCA* while bars and horizontal dashed line show ± 1 s.d. The protein times series shown in **(C)** illustrate the two dynamic regimes reached when changing the diffusion coefficient in the uniform configuration: stationary state (**(C1)**, *D*=1.67×10^−3^) or oscillations with low (**(C2)**, *D*=1.67×10^−2^) or high (**(C3)**, *D*=1.67×10^−1^) frequency (note the difference in time scale in **C3**). All other parameters were according to the standard set defined in the Methods section.

Figure 6A shows the evolution of the average FZCA when the degree of demixing changes in 3D. Just like in 2D (Figure 4), the dynamics remains oscillatory for the clustered configuration whatever the degree of demixing, while a space-induced bifurcation occurs at large demixing (small *r*/*R* values) for the segregated configuration: the dynamics changes from oscillatory to stationary when demixing increases. Note that the effect of increasing degrees of demixing is less marked in 3D than in 2D (Figure 4), as a result of the changes in the properties of diffusion. However, like in 2D, the space induced bifurcation is mostly governed by the spatial range of the proteins and mRNA, i.e. the average distance travelled before degradation (*τ*_*M*_*D* and *τ*_*P*_*D*). In Figure 6B, we thus manipulate the spatial range by changing the diffusion coefficient *D* of the mRNA and of the proteins. Since we keep the lifetime of both species constant in those simulations, the smaller the diffusion coefficient, the smaller the spatial range. For very large values (*D*>0.1 a.u. ^2^/MC time step), the spatial range is so large that each mRNA and each protein can explore most of the whole accessible space before degradation, thus yielding effective perfect mixing, even if the gene positions are not well mixed. In this case, the three types of gene configurations yield oscillatory dynamics (Figure 6C3). Note that in this case, the regime is oscillatory with high frequency (period of roughly 50,000 MC time steps). The low FZCA value in Figure 6B for *D*>0.1 thus reflects those low-period oscillations, not noisy fluctuations around a stationary state. In a qualitative way, this is a similar phenomenon to labels (*a*^′^−*c*^′^) in Figure 5A: when the spatial range is large (either because the degradation rate is low or the diffusion coefficient large), spatially explicit simulation converge to the predictions of the mass action kinetics and whatever the spatial configuration of the genes, the dynamical regime is oscillatory.

When the diffusion coefficient decreases, the dynamics in those 3D simulations exhibits two phases: for intermediate diffusion coefficients, i.e. $D \gtrsim 10^{-2}$ a.u. ^2^/MC time step (Figure 6B) the regime remains oscillatory for all the gene configurations (Figure 6B), but the period of the oscillations increases to recover the values observed for the oscillatory regimes in 2D above (i.e. close to 200,000 time steps, compare Figure 6C3 with Figure 3A). When the diffusion coefficient decreases further (*D*≲2×10^−2^ a.u. ^2^/MC time step), the behavior depends on the spatial configuration of the genes. With the clustered configuration, the oscillatory regime persists (with low frequency), at least in the limit of the values of *D* that we tested. With the uniform or segregated configurations however, the dynamics exhibit a qualitative change as *D* decreases below 2×10^−2^, switching from an oscillatory regime (Figure 6C2) to a stationary one where all species fluctuate around a constant steady state (Figure 6C1). Therefore, this is a further illustration that space-induced bifurcations are also observed in 3D when the spatial range decreases. In agreement with our 2D results above, the segregated configuration appears to be more sensitive than the uniform one: the oscillatory-stationary bifurcation seems to necessitate less reduction of the diffusion coefficient in the segregated configuration than in the uniform one. This observation is a further example that the dynamic regime in our system is strongly controlled by the gene positions in space.

## Discussion

Our goal in this study was to investigate whether space can influence the temporal dynamics of repressilator circuits i.e. 3-gene repression-based transcriptional regulatory loops, that exhibit prototypical spontaneous oscillations in certain ranges of kinetic parameters (transcription/translation rate, species lifetimes). We used spatially-explicit stochastic individual-based modelling to simulate repressilator circuits in 2D and 3D, with various degrees of demixing of the gene positions in space. Our main finding is that variations of some spatial parameters (degree of demixing of the genes, spatial range of the mRNA and proteins) can have dramatic effects on the system dynamics, switching it from the spontaneous oscillatory regime to a stationary regime where each species fluctuates around constant values. This effect is similar to the bifurcations along the kinetic parameters that are usually evoked to explain the appearance of the oscillatory regime in those systems. We thus referred to it as a space-induced bifurcation. Our study therefore indicates that spatial parameters should be considered as additional bifurcation dimensions to the kinetic parameters to predict the dynamics of those systems. This conclusion strongly supports the idea that the spatial organization of the molecular actors of transcriptional networks is crucial for the dynamics of gene expression.

The possibility that the positions in space of the elements of intracellular biochemical systems may control their dynamics has already been suggested in previous works (see e.g. [24-26,44-46]). In the specific case of the dynamics of gene expression, the study of the influence of space on the dynamics of the expression of an isolated gene has recently started to be explored with computational or theoretical approaches. The main conclusion was that diffusion of mRNAs and proteins and the spatial correlations created by the coupling with reaction, can strongly increase the fluctuations of gene expression, i.e. noise [40,41,47]. Our results is a significant advance in this problem since we consider gene networks (albeit small ones), not a single isolated gene and we show that the alterations of the dynamics due to spatial parameters in those systems may be qualitative: beyond changing the mean value or the fluctuations, they may even alter the global regime of the dynamics (stationary vs oscillatory).

From our simulation results, two major experimental predictions emerge concerning the dynamics of 3-gene repression-based transcriptional ring networks. If each of the 3 genes is present in a single copy (see Figure 5B, *G*_*T*_=1), we expect the dynamics to be stationary in most cases, except if each of the repressor gene is located very close to its repressed target gene (i.e. the clustered scenario). In other words, we predict that spontaneous oscillations may be difficult to obtain in networks with a single copy of each gene. Moreover, our study suggests that the spatial range of the proteins and mRNAs, i.e. their Kuramoto length (the typical distance they travel before degradation), is a major determinant of the system dynamics. The precise location of the genes is likely to control the dynamics when the spatial range (i.e. the product of the protein or mRNA lifetime and its diffusion coefficient) is not too large. For large spatial ranges (i.e. Figure 5A, 1/*τ*_*M*_=0.005 or Figure 6B, *D*=0.167), the expected regime agrees with the prediction of mass action kinetics, i.e. spontaneous oscillations with the kinetic parameters we used. Therefore, *in vivo*, space-induced bifurcations should be significant in systems where the lifetimes and/or the diffusion coefficient of the mRNAs and/or proteins are limited.

Estimating the spatial range of intracellular proteins *in vivo* is still a challenge for experimental biology. Based on the measured diffusion coefficient of Fus3 MAP kinase in the yeast [48] or that of GFP in *E. coli* [49], Cottrell et al. [41] obtained coarse estimates leading to the conclusion that cytoplasmic proteins should have spatial ranges that are much larger than the cell itself. This leads to the widespread opinion that the spatial distribution of cytoplasmic proteins in the cell is uniform (well-mixed). However, even in bacteria like *E. coli*, this idea is questionable. First, consistent recent experimental evidence suggest that many proteins adopt localized distribution inside the cell, in opposition to a well-mixed situation [50-53]. The molecular actors of gene expression in *C. crescentus* or *E.coli* may even present a very specific spatially-organized intracellular structure [20], with, in particular, chromosomally-expressed mRNAs that exhibit very low diffusion coefficients [19]. Another recent and very symbolic example is LacI, the repressor of the *lac* operon. Direct measurements of the diffusion coefficient of LacI in living *E.coli* cells indicate rapid diffusion (*D* of the order or 0.1–1 μ*m*^2^/s) and consequently suggest a very large spatial range [54]. However, recent measurement of the steady-state distribution of LacI inside living *E.coli* revealed that depending on the location of the *lacI* gene on the bacterial chromosome, the distribution of the LacI protein can either be homogeneous or highly inhomogeneous and mostly localized [55]. In fact, the diffusion of proteins and mRNAs in living cells, even in simple and small cells like *E.coli* is far from a simple Brownian motion. This may lead to violations of the hypotheses that underly coarse estimations of the spatial range. First, for macromolecules that interact with DNA, the diffusion process itself is composite (facilitated diffusion) because part of it occurs in 3D in the bulk and part of it as a restricted sliding movement along the DNA molecule [56-58]. Moreover the bacterial cytoplasm itself does not present the usual properties of a liquid. It is a complex, extremely crowded and dense medium that strongly protein and mRNA mobility in a spatially non-homogeneous way [11-15]. How exactly those complex properties arise from the intracellular elements and how exactly they alter molecule mobility is still unclear [15], but theoretical arguments indicate that such macromolecular crowding may strongly affect the dynamics of gene expression [47,59]. We conclude that, in spite of the rather large values measured for the diffusion coefficient of some proteins or mRNAs, the physical nature of the cytoplasm is likely to considerably restrict the spatial range of proteins and mRNAs. Therefore, one would generically expect that space-induced bifurcations may be significant in living cells.

One possibility that we have left unexplored in the present work is that of an inverse transition: starting from a region in the parameter space where mass action kinetics predict a stable steady state (i.e. the white region in Figure 1B), can oscillations be induced by manipulation of some spatial parameter? We first stress that the observed shunting down of oscillations resulting from spatial segregation is not exactly a return to the steady state of the mass action kinetics, since in the latter, all three proteins are predicted to be expressed in equal amounts whereas in the former, one of the proteins is often expressed at a much lower level than the others. In previous works [24-26], we have showed that in the absence of bifurcation in the mass action kinetics, the unique equilibrium remains stable, globally and asymptotically. However, when a bifurcation does exist in the mass action kinetics approximation, the question remains open, especially when the parameters are very close to the bifurcation of the mass action kinetics system.

## Conclusion

Our model for gene expression is limited to the main processes implied in gene expression and is limited to a 3-gene network. We think that the basic mechanisms underlying space-induced bifurcations are simple enough that they should still be effective in other systems, in particular, in transcriptional regulation networks comprising larger numbers of gene types or positive regulation (activators). Likewise, we expect our conclusions to be robust to increasing molecular details and more realistic modelling of each subprocess (RNA polymerase, initiation, elongation, termination, ribosomes…). Therefore, our results bring a strong support to the view that the spatial organization of the molecular actors of transcriptional networks is crucial for the dynamics of gene expression. In a synthetic biology perspective, they suggest that the spatial localisation of the synthetic genes should be factored out in the global strategy used to shape the dynamics of the synthetic network to be inserted in the cell.

## Additional files

Additional file 1
**Figure S1.** The effect of the spatial configuration of the genes disappears when the lifetime of mRNAs is very large (2D simulations). The time courses of the total number of proteins (*P*_0_ in blue, *P*_1_ red and *P*_2_ green) in the reaction space were obtained using spatially-explicit stochastic individual-based simulations in 2D. The spatial configuration of the genes corresponded to the **(a’)** uniform, **(b’)** clustered (*r*=3) or **(c’)** segregated (*r*=3) configuration, corresponding to the FZCA points labelled (a’), (b’) and (c’), respectively in Figure 5A. The mRNA degradation rate was set to 0.005 (MC time step) ^−1^. All other parameters were according to the standard set defined in the Methods section.

Additional file 2
**Figure S2.** Dynamics with a unique gene copy per type (2D simulations).The time courses of the total number of proteins (*P*_0_ in blue, *P*_1_ red and *P*_2_ green) in the reaction space were obtained using spatially-explicit stochastic individual-based simulations in 2D. The spatial configuration of the genes corresponded to the **(a”)** uniform, **(b”)** clustered (*r*=3) or **(c”)** segregated (*r*=3) configuration, corresponding to the FZCA points labelled (a”), (b”) and (c”), respectively in Figure 5B. Here, *G*_*T*_=1, while all other parameters were according to the standard set defined in the Methods section.
