# Supplementary figures and images for: Space-induced bifurcation in repression-based transcriptional circuits
Source: BMC Syst Biol. 2014 Nov 8;8:125. doi: 10.1186/s12918-014-0125-z (PMC4233083; doi:10.1186/s12918-014-0125-z)

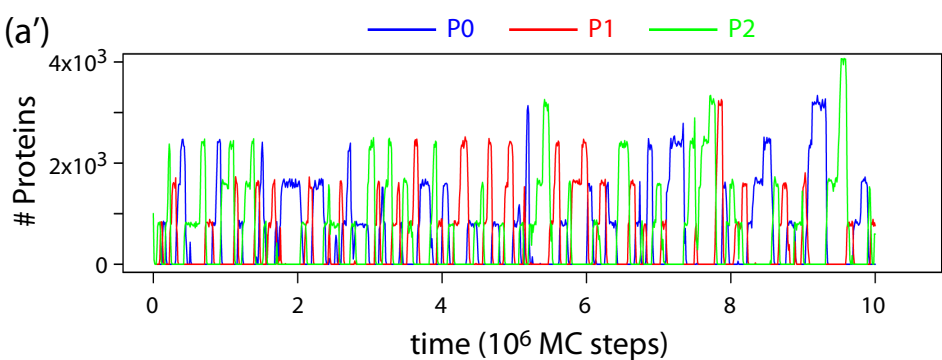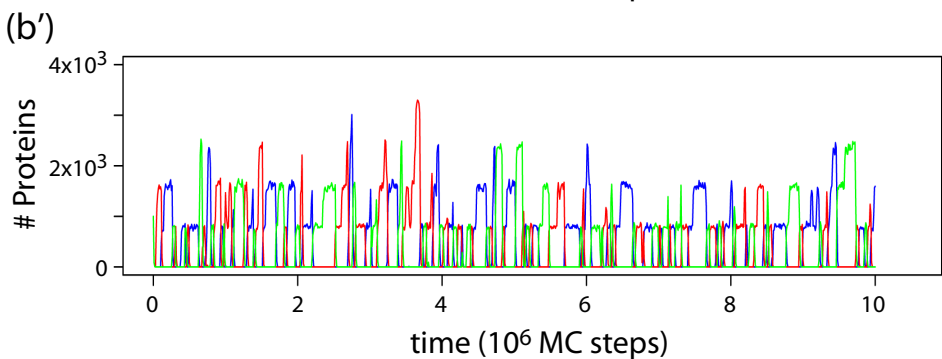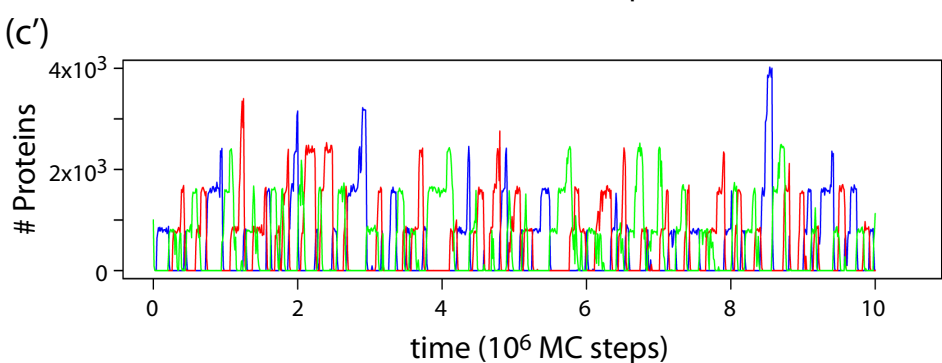

Supplement: Additional file 1 — Figure S1. The effect of the spatial configuration of the genes disappears when the lifetime of mRNAs is very large (2D simulations). The time courses of the total number of proteins (P 0 in blue, P 1 red and P 2 green) in the reaction space were obtained using spatially-explicit stochastic individual-based simulations in 2D. The spatial configuration of the genes corresponded to the (a’) uniform, (b’) clustered (r=3) or (c’) segregated (r=3) configuration, corresponding to the FZCA points labelled (a’), (b’) and (c’), respectively in Figure 5A. The mRNA degradation rate was set to 0.005 (MC time step) −1. All other parameters were according to the standard set defined in the Methods section. [file 12918_2014_125_MOESM1_ESM.pdf]

(a'')

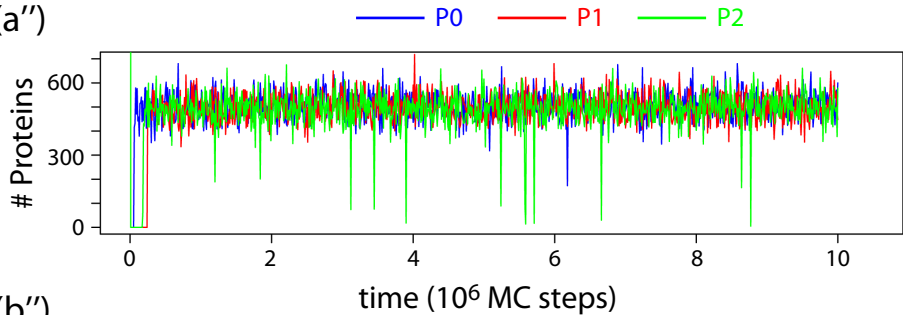

(b'')

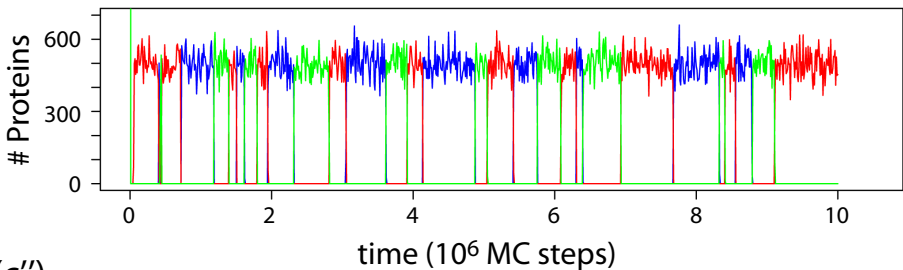

(c'')

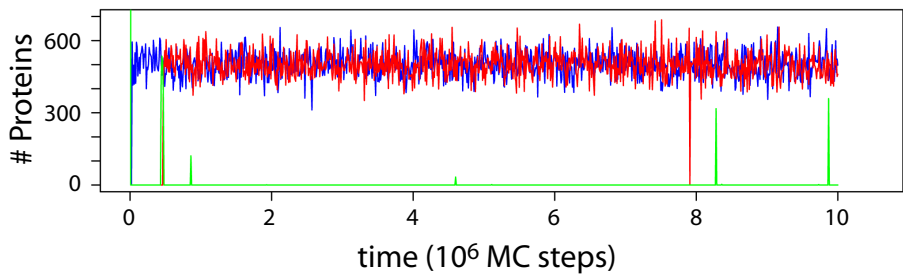

Supplement: Additional file 2 — Figure S2. Dynamics with a unique gene copy per type (2D simulations).The time courses of the total number of proteins (P 0 in blue, P 1 red and P 2 green) in the reaction space were obtained using spatially-explicit stochastic individual-based simulations in 2D. The spatial configuration of the genes corresponded to the (a”) uniform, (b”) clustered (r=3) or (c”) segregated (r=3) configuration, corresponding to the FZCA points labelled (a”), (b”) and (c”), respectively in Figure 5B. Here, G T=1, while all other parameters were according to the standard set defined in the Methods section. [file 12918_2014_125_MOESM2_ESM.pdf]
